# Liver-Directed Therapy in Neuroendocrine Neoplasms Metastatic to Both Liver and Bone

**DOI:** 10.3390/jcm12247646

**Published:** 2023-12-13

**Authors:** Kristen E. Limbach, Kelly M. Mahuron, Aaron T. Scott, Philip H. G. Ituarte, Gagandeep Singh

**Affiliations:** Department of Surgery, City of Hope National Medical Center, Duarte, CA 91010, USA; klimbach@tulane.edu (K.E.L.); kmahuron@coh.org (K.M.M.); aascott@coh.org (A.T.S.); pituarte@coh.org (P.H.G.I.)

**Keywords:** neuroendocrine tumors, liver-directed therapy, bone metastasis

## Abstract

Bone metastases from gastroenteropancreatic neuroendocrine neoplasms (GEPNENs) have been associated with poor prognosis, but it is unclear whether patients with concurrent bone metastases who receive liver-directed therapy (LDT) would derive survival benefit. The California Cancer Registry dataset, merged with data from the California Office of Statewide Health Planning and Development, was used to perform a retrospective study of GEPNENs metastatic to both liver and bone between 2000 and 2012. A total of 203 patients were identified. Of these, 14.8% underwent LDT after bone metastasis diagnosis, 22.1% received LDT prior to that diagnosis, and 63.1% never received LDT. The median overall survival from the time of bone metastasis diagnosis was significantly longer in those that received LDT after diagnosis when compared with those that never received LDT (*p* = 0.005) and was not significantly different from the median overall survival of those that had received LDT prior to diagnosis (*p* = 0.256). LDT may still be associated with improved survival even after a diagnosis of bone metastasis.

## 1. Introduction

Gastroenteropancreatic neuroendocrine neoplasms (GEPNENs) are an uncommon and heterogeneous group of neoplasms [1,2] that frequently metastasize, often to the liver [2]. Traditionally, bone metastases were considered to be exceptionally rare [3,4], with foregut and hindgut gastrointestinal primary tumors representing the most common sites of origin and midgut primaries less likely to spread to bone [5]. However, one small autopsy study found skeletal metastases in 42% of subjects [6], and more recent series show an increased frequency of diagnosis with a prevalence of up to 12–13% [7,8]. This increased rate of detection in the modern era may be in part due to improved imaging options, such as bone scintigraphy [8,9], and the development of more sensitive functional imaging [10,11,12]. In particular, ^68^Gallium-DOTATATE positron emission tomography has been shown to influence changes in the management of a significant percentage of patients [13,14] and improve the detection of bone metastases in patients with neuroendocrine neoplasms [12,15]. Thus, the prevalence of neuroendocrine bone metastases may be much higher than previously suspected, and rates of detection may continue to rise with the increasingly widespread use of ^68^Gallium-DOTATATE positron emission tomography.

The detection of bone metastases may have substantial implications for patient management, as bone metastases have been associated with a worse prognosis [4,7]. In the series by Van Loon et al. published in 2015, patients with gastrointestinal carcinoid tumors metastatic to both liver and bone had a significantly lower median overall survival (OS) than those with liver metastases only (47.8 versus 99.5 months, *p* < 0.001) [7], and another study noted a mortality rate of 42% in the year following diagnosis of bone metastases [8]. Unfortunately, treatment options for neuroendocrine bone metastases are few, and the comparatively uncommon nature of this disease process has limited the availability of prospective data [16]. Current recommendations include palliative treatment of bone pain with analgesics and radiation therapy, with surgical resection reserved for cases of skeletal instability [16,17]. However, it has been noted that the major cause of death in patients with metastatic GEPNENs is liver failure when liver metastases are present, even in patients with extrahepatic metastases [18,19]. Unsurprisingly, in GEPNENs metastatic to the liver, liver-directed therapy, including surgical resection, ablation, and embolization, has been associated with a significant improvement in survival [18,20,21,22]. Given this association, it has been suggested that LDT may be still be indicated in patients with pancreatic neuroendocrine tumors metastatic to both liver and extrahepatic sites [18], and the question remains whether LDT would be associated with improved survival for other GEPNENs with extrahepatic metastases.

The aim of this study is to use a population-based approach to examine the role of LDT in patients with neuroendocrine neoplasms diagnosed with both liver and bone metastases. Given that hepatic failure is a substantial cause of mortality in this population, we hypothesize that improved survival will be seen in patients who receive liver-directed treatment, even in the context of concurrent extrahepatic metastases.

## 2. Materials and Methods

### 2.1. Data

The California Cancer Registry (CCR) dataset was merged with the California Office of Statewide Health Planning and Development (OSHPD) discharge data and used to perform a retrospective study of patients diagnosed with GEPNENs metastatic to both liver and bone. The CCR is a statewide repository of cancer records and includes patient demographic data, as well as tumor site, histology, stage, treatment, and follow-up information. The OSHPD database contains admission and discharge data for all nonfederal acute care facilities in the state of California, in both in- and outpatient care settings. Diagnoses and procedures were coded as designated by the International Classification of Disease 9th Edition (ICD-9-CM). Approval for use of the CCR-OSHPD-linked data was obtained from both the City of Hope Institutional Review Board and the California Committee for the Protection of Human Subjects.

### 2.2. Patients

The CCR-OHSPD-linked database was queried to identify patients older than 18 years with a diagnosis of neuroendocrine neoplasm of gastrointestinal or pancreatic origin between 2000 and 2012 and documented metastases to both liver and bone. Neuroendocrine neoplasm diagnosis was confirmed using the ICD-0-3 histology codes 8240–8246, 8249, and 8150–8152. Gastrointestinal or pancreatic origin was determined by site-specific codes including the stomach (C160–C166, C168–C169), the small intestine (C170–C173, C178–C179), the colorectal area including the appendix (C180–C189, C199, C209), and the pancreas (C250–C254, C257–C259). Liver and bone metastatic status was determined by ICD-9 diagnosis codes of 197.7 (malignant neoplasm of liver, secondary), 209.72 (secondary NET of liver), 198.5 (secondary malignant neoplasm of bone and bone marrow), and 209.73 (secondary NET of bone). Other sites of metastasis were determined in a similar fashion. The modalities of liver-directed therapy included were surgical resection, ablation, and embolization including both transarterial chemoembolization and transarterial radioembolization. Patients without a histologic confirmation of diagnosis, patients with a diagnosis on death/autopsy or within 30 days, patients lacking inpatient or outpatient records, and patients under 18 years of age were excluded.

### 2.3. Statistical Analysis

The primary outcome of this study was overall survival (OS). Patient characteristics including demographics and treatment were compared between groups using Student’s *t*-test for continuous variables, the Pearson chi-square test for categorical variables, and the Mann–Whitney U test for ordinal variables. OS was determined using the method of Kaplan and Meier and compared using the log-rank test. Cox regression was used to estimate treatment effect with stratification by treatment modality. The level of significance was set at *p* = 0.05. All statistical analysis was performed using SPSS version 28.0, Armonk, NY, USA.

## 3. Results

**Figure 1:**
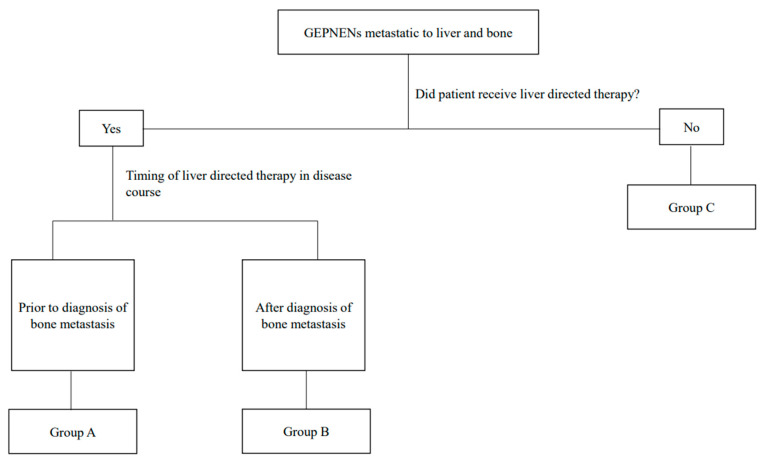
Schema of study design.

Two hundred and three patients that met the inclusion criteria were identified in the CCR-OSHPD-linked database. Seventy-five (36.9%) of these patients underwent LDT including resection, ablation, or embolization; of these 75, 45 (22.1%) received LDT prior to a diagnosis of bone metastasis (Group A), and 30 (14.8%) received LDT after that diagnosis (Group B). The remaining one hundred and twenty-eight (63.1%) patients never received LDT (Group C) (Figure 1). Of the 203 patients included, 18 had a stomach primary tumor (8.9%), 88 (43.3%) had a pancreatic primary, 33 (16.3%) a small bowel primary, and 64 (31.5%) a colorectal or appendiceal primary. Of those patients in Group B, who all received liver-directed therapy after a diagnosis of bone metastases, the vast majority underwent transarterial chemoembolization (TACE) (29 patients, 96.7%). Patient demographics are shown in Table 1.

A comparison of patients who never received LDT (Group C) with those who received LDT after a diagnosis of bone metastases (Group B) revealed no significant difference in age (59.6 vs 54.7 years, *p* = 0.080), race/ethnicity (*p* = 0.472), Charlson comorbidity score (*p* = 0.423), the proportion of patients with at least one additional site of metastasis outside of bone or liver at the time of bone metastasis diagnosis (33.3% vs. 51.1%, *p* = 0.462), or tumor grade (*p* = 0.343). However, a comparison of patients in Group B with those in Group A did reveal a significantly higher Charlson comorbidity score (Charlson comorbidity score ≥2 in 60% vs. 24.4%, *p* = 0.001), while there were no significant differences in age (54.7 vs. 54.0 years, *p* = 0.807), race/ethnicity (*p* = 0.178), the proportion of patients with at least one additional site at time of bone metastasis diagnosis (33.3% vs. 40.6%, *p* = 0.129), or grade (*p* = 0.449). Of note, patients in Group B were also more likely to undergo radiation therapy than those in Group A (33.3% vs. 8.9%, *p* = 0.008). There was no significant difference between Groups A and B in the percentage of patients who had additional sites of metastasis outside of liver and bone at the time of their LDT (36.7% vs. 20.0%, *p* = 0.110). The time interval between the diagnosis of liver and bone metastases was significantly shorter in patients who received LDT after the diagnosis of skeletal involvement (Group B) than in those who received it before (Group A), with a median time of 0 months between diagnoses versus 19.9 months (*p* < 0.001). There was no significant difference in the time interval between diagnoses when compared with patients in Group C (0 vs. 0 months, *p* = 0.678).

**Figure 2:**
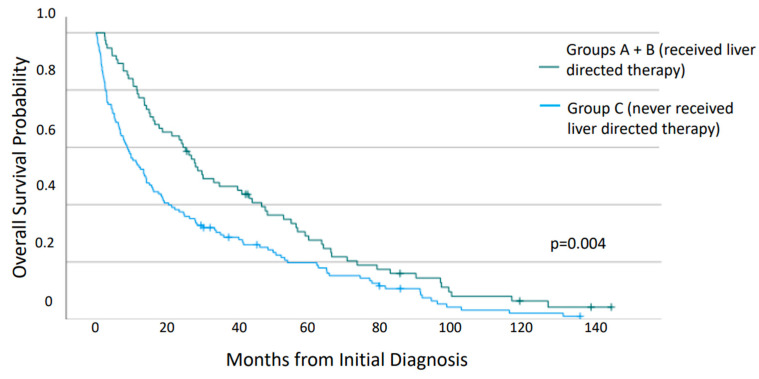
Overall survival of patients from time of diagnosis with gastroenteropancreatic neuroendocrine neoplasms metastatic to both liver and bone, compared according to whether liver-directed therapy was given.

Analysis of survival from the time of initial diagnosis of neuroendocrine neoplasm (Figure 2) revealed a significantly increased median OS for patients who received LDT at any time in their disease course (29.9 vs. 13.5 months, *p* = 0.004). Of those who received LDT (Groups A and B), there was no significant difference in median OS between those that had additional sites of metastasis outside of liver and bone at the time of their LDT and those that did not (25.1 vs. 34.6 months, *p* = 0.889). However, those that underwent surgical resection or ablation had significantly improved survival when compared with those that underwent embolization therapy or no LDT (median OS 63.2 vs. 23.3 vs. 13.5 months, *p* < 0.001). The hazard ratio was 0.40 and 0.81 for the patients who had undergone surgical resection/ablation and embolization, respectively.

**Figure 3:**
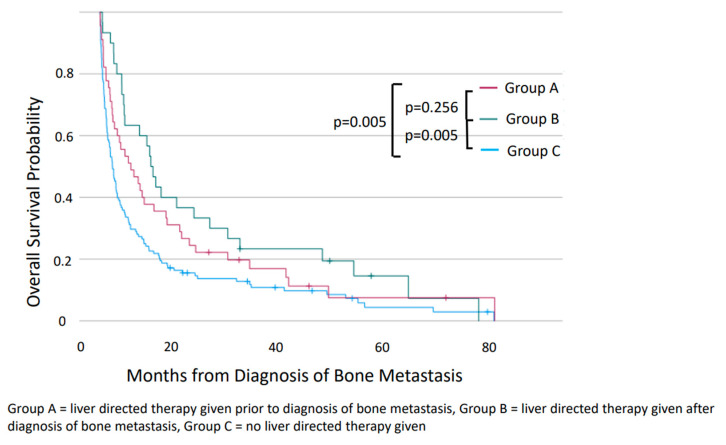
Overall survival of patients from diagnosis of bone metastasis of patients with gastroenteropancreatic neuroendocrine neoplasms metastatic to both liver and bone, compared between Groups A, B, and C.

A comparison of the three groups revealed the most favorable prognosis in patients in Group A, with a median OS of 47.4 months compared with 18.6 months for those in Group B (*p* = 0.006). There was no difference in median OS between Groups B and C (*p* = 0.688). An analysis of survival when calculated from the time of diagnosis of bone metastases revealed significant differences in median OS (Figure 3). The median OS for patients in Group B was significantly longer than those in Group C (9.3 vs. 2.3 months, *p* = 0.005) and was similar to that of those in Group A (9.3 vs. 5.6 months, *p* = 0.256).

## 4. Discussion

The findings of this study suggest that liver-directed therapy in patients with neuroendocrine neoplasms metastatic to the liver is associated with improved overall survival and may provide benefit even if administered after they have also been diagnosed with bone metastases. Although the comparatively short survival of patients with bone metastases in this study is consistent with the prior literature suggesting a poor prognosis [7], those in Group B, who underwent LDT after their diagnosis of bone metastases, did have a significantly longer median OS than those who never underwent LDT. Furthermore, OS when calculated from the time of diagnosis of bone metastases was not significantly different between Groups A and B, suggesting that the timing of LDT is less important in overall prognosis than whether the patient receives it. This finding is consistent with the previous literature that has identified liver failure as the predominant cause of mortality in this population [18,19]; given that patient survival is driven by the liver rather than other sites of metastasis, it follows that survival is improved in patients who receive treatment directed at decreasing the burden of disease in the liver.

These survival analyses may have been impacted by several factors, and it is difficult to quantify the extent to which they may have contributed to the reported findings. Firstly, the interval between the development of liver and bone metastases was significantly longer in those in Group A. This is notable in that a longer lead time prior to the development of bone metastases may suggest a more indolent biology for patients in this group, even though histologic grade distribution was not significantly different. However, exact Ki-67 values were not available in this dataset, which may have limited this comparison, and as extrahepatic metastatic sites are not routinely sampled, there is no way to include the Ki-67 or the grade of the bone metastases themselves, which can vary from that of the primary tumor. Thus, there may be a biological variable that could not be well examined. A theoretical biological difference between these patient populations (specifically Groups A and B) may also explain the lack of survival improvement for Group B when examined from the time of their diagnosis. In addition, the modality of LDT may have played a role in the observed outcomes as surgical resection or ablation was associated with improved survival over transarterial therapy. It is difficult to specifically compare these populations given that granular data regarding resectability status are not available, but it is notable that Group B was universally treated with transarterial therapy, which may have contributed to poorer survival.

Further research will be necessary to examine comparative survival after treatment with peptide receptor radionuclide therapy (PRRT), now that PRRT has become more common in the treatment of patients with extrahepatic metastases [23,24]. The population included in this study was treated prior to FDA approval of PRRT in the United States, and the advent of PRRT may have a significant impact on this population, particularly those with multiple sites of metastasis [25]. Additional sites of metastasis outside of liver and bone did not appear to have a significant impact on survival in this study but may influence providers in their choice of treatment, which could then have downstream effects on survival.

These findings are limited by multiple additional factors. First and foremost, as has been the case for other similar studies, the comparative rarity of this disease process results in a small study size. Furthermore, the data available from this population-based dataset lack granularity regarding the resectability status of liver metastases, the burden of metastatic disease, and the cause of death. It should be noted that details of the exact Ki-67 as well as a further description of the tumors included are not available in this dataset. However, the comparison of survival from the time of diagnosis of bone metastases provides a clinically relevant viewpoint, and the findings suggest that at a population level, there may be benefit to offering LDT to this population.

## 5. Conclusions

This study suggests that LDT may provide a survival benefit in patients with GEPNENs metastatic to the liver even after a diagnosis of bone metastases. Further study is warranted to determine whether certain patient populations within this group derive particular benefit and to establish the role of treatment modality.

## Figures and Tables

**Table 1:** Demographic characteristics of included patients, grouped by whether patient received liver-directed therapy and the timing of that in relation to their diagnosis of bone metastasis.

Factors	Group A: Liver-Directed Therapy Prior to Diagnosis of Bone Metastasis *n* (%)	Group B: Liver-Directed Therapy after Diagnosis of Bone Metastasis *n* (%)	Group C: No Liver-Directed Therapy *n* (%)
Patients	45 (22.1)	30 (14.8)	128 (63.1)
Age in years, mean ± SD	54.0 ± 12.3	54.7 ± 14.2	59.6 ± 13.5
Male	24 (53.3)	16 (33.3)	66 (51.6)
Race/Ethnicity			
Caucasian	32 (71.1)	15 (50)	74 (57.8)
Other	13 (28.9)	15 (50)	54 (42.2)
Charlson Comorbidity score			
≥2	11 (24.4)	18 (60)	57 (44.5)
Primary tumor site			
Pancreas	20 (44.4)	11 (36.7)	57 (44.5)
Colorectal	12 (26.7)	13 (43.3)	39 (30.5)
Additional sites of metastasis			
Lung	15 (33.3)	11 (36.7)	33 (25.8)
Peritoneum and/or brain	14 (31.1)	11 (36.7)	33 (25.8)
Grade III histology	24 (53.3)	20 (66.7)	87 (68.0)
Received embolization therapy	40 (89.9)	30 (100)	-
Received chemotherapy	25 (55.6)	22 (73.3)	51 (39.8)

SD = standard deviation.

## Data Availability

Data are available from the California Cancer Registry, found at https://www.ccrcal.org/retrieve-data/data-for-researchers/how-to-request-ccr-data/.
